# Safety and Immunogenicity of a Plant-Produced Recombinant Hemagglutinin-Based Influenza Vaccine (HAI-05) Derived from A/Indonesia/05/2005 (H5N1) Influenza Virus: A Phase 1 Randomized, Double-Blind, Placebo-Controlled, Dose-Escalation Study in Healthy Adults

**DOI:** 10.3390/v4113227

**Published:** 2012-11-19

**Authors:** Jessica A. Chichester, R. Mark Jones, Brian J. Green, Mark Stow, Fudu Miao, George Moonsammy, Stephen J. Streatfield, Vidadi Yusibov

**Affiliations:** Fraunhofer USA, Center for Molecular Biotechnology, Newark, DE 19711, USA; Email: jchichester@fraunhofer-cmb.org (J.A.C.); mjones@fraunhofer-cmb.org (R.M.J.); bgreen@fraunhofer-cmb.org (B.J.G.); mstow@fraunhofer-cmb.org (M.S.); fmiao@fraunhofer-cmb.org (F.M.); gmoonsammy@fraunhofer-cmb.org (G.M.); sstreatfield@fraunhofer-cmb.org (S.J.S.); vyusibov@fraunhofer-cmb.org (V.Y.)

**Keywords:** influenza A, H5N1, recombinant vaccine, hemagglutinin, plant-produced vaccine

## Abstract

Recently, we have reported [1,2] on a subunit influenza vaccine candidate based on the recombinant hemagglutinin protein from the A/Indonesia/05/2005 (H5N1) strain of influenza virus, produced it using ‘launch vector’-based transient expression technology in *Nicotiana benthamiana*, and demonstrated its immunogenicity in pre-clinical studies. Here, we present the results of a first-in-human, Phase 1 randomized, double-blind, placebo-controlled study designed to investigate safety, reactogenicity and immunogenicity of three escalating dose levels of this vaccine, HAI-05, (15, 45 and 90 µg) adjuvanted with Alhydrogel^®^ (0.75 mg aluminum per dose) and the 90 µg dose level without Alhydrogel^®^. Vaccine was administered intramuscularly in two injections three weeks apart to healthy adults of 18–49 years of age. At all dose levels the vaccine was generally safe and well tolerated, with no reported serious adverse events or dose-limiting toxicities. Mild local and systemic reactions were observed in all vaccine dose groups and the placebo group and their occurrence was not dose related. The incidence rates were higher in the groups receiving vaccine with Alhydrogel^®^. The immune response elicited by the HAI-05 vaccine was variable with respect to both hemagglutination-inhibition and virus microneutralization antibody titers, with the highest responses observed in the 90 µg unadjuvanted group.

## 1. Introduction

Infections with influenza viruses cause a serious respiratory disease, due to which three to five million cases of severe illness are recorded worldwide annually, with 250,000 to 500,000 deaths [3]. In addition to seasonal influenza outbreaks, occasional influenza pandemics can arise at any time when influenza A virus containing a novel hemagglutinin (HA) subtype is introduced and effectively transmitted among humans. In 1997, a highly pathogenic avian influenza strain of subtype H5N1 caused widespread disease in poultry in Hong Kong. The virus spread rapidly from 2004 onwards and has now caused serious poultry disease outbreaks in several Asian countries, Europe and Africa. More than 500 individuals have been infected with H5N1 viruses with a mortality rate of 50–60%. Fortunately, almost all reported human cases have had close contact with infected birds, with very rare instances of presumed human to human transmission. Nevertheless, considerable concern for human health remains, not only because of the severity of human cases, but also because the virus could undergo adaptive mutation or re-assortment and obtain the ability to spread efficiently among humans [4,5,6]. Should these events occur, the H5N1 and/or other novel viruses could have the potential to cause a pandemic exhibiting a very aggressive clinical progression with devastating health and economic consequences globally. Such a scenario underscores the urgent need for rapid and robust influenza vaccine production methods to better prevent and mitigate the effects of pandemics in the future.

The potential of plants as a platform for the production of subunit vaccines and therapeutic proteins has been recently demonstrated [7]. We have developed an approach for transient expression of target proteins in healthy *Nicotiana benthamiana* using a Tobacco mosaic virus-based ‘launch’ vector system [8] and demonstrated its utility by producing vaccine antigens derived from *Bacillus anthracis* [9], *Yersinia pestis* [10], and seasonal and pandemic strains of influenza virus [1,11,12]. 

Recently, we have developed a recombinant HA-based vaccine, HAI-05, for the prevention of disease caused by the novel A/Indonesia/05/2005 (H5N1) strain of influenza A virus, and produced this target in *N. benthamiana* at pilot plant scale under current Good Manufacturing Practice (cGMP) guidelines [2]. Pre-clinical studies have demonstrated immunogenicity of HAI-05 in mice, rabbits and ferrets [2] and prompted further investigation of this vaccine candidate in humans. Here, we report the results of a Phase 1 study conducted to determine safety, reactogenicity and immunogenicity of three escalating dose levels of HAI-05 (15, 45 and 90 µg) adjuvanted with Alhydrogel^®^ and the 90 µg dose level without Alhydrogel^®^, delivered intramuscularly to healthy adults of 18–49 years of age.

## 2. Results and Discussion

This first-in-human, Phase 1 randomized, double-blind, placebo-controlled clinical trial was conducted to evaluate safety, reactogenicity and immunogenicity of two doses of the HAI-05 vaccine delivered at three escalating dose levels—15, 45 and 90 µg adjuvanted with Alhydrogel^®^ and 90 µg without Alhydrogel^®^—in healthy adults 18–49 years of age.

### 2.1. Study Population

Of the 100 subjects who were randomized and treated, all received both scheduled vaccinations (Table 1). The mean age of participants was 30–32 years for the active dose groups and approximately 34 years for the placebo group. The proportion of female subjects was higher in the active dose groups (55–72%), whereas equal numbers of female and male subjects were enrolled in the placebo group. Most subjects in all groups were white (65–85% in the active dose groups and 86% in the placebo group) (Table 2). 

**Table 1:** Subject disposition.

	Adjuvanted HAI-05			Unadjuvanted HAI-05	Placebo
	15 µg	45 µg	90 µg	90 µg	
	n (%)	n (%)	n (%)	n (%)	n (%)
All subjects treated	18 (100)	20 (100)	20 (100)	20 (100)	22 (100)
Intent-to-treat population ^a^	18 (100)	20 (100)	20 (100)	20 (100)	22 (100)
Per-protocol population ^b^	18 (100)	20 (100)	20 (100)	20 (100)	22 (100)
Completed the study	16 (88.9)	20 (100)	19 (95.0)	20 (100)	21 (95.5)
Discontinued from the study ^c^	2 (11.1)	0	1 (5.0)	0	1 (4.5)

^a^: The “Intent-to-treat” population included all subjects who received one dose of vaccine.

^b^: The “Per-protocol” population included all subjects who received two doses of vaccine and had a complete set of serum specimens for antibody determination.

^c^: Reason for discontinuation: lost to follow-up.

**Table 2:** Demographic characteristics.

		Adjuvanted HAI-05			Unadjuvanted HAI-05	Placebo
		15 µg	45 µg	90 µg	90 µg	
		N = 18	N = 20	N = 20	N = 20	N = 22
**Female, n (%)**		13 (72.2)	11 (55.0)	12 (60.0)	12 (60.0)	11 (50.0)
**Age (years)**						
	n	18	20	20	20	22
	Mean	31.8	31.1	32.0	30.1	34.1
	SD	8.3	9.2	9.6	7.8	9.5
	Median	30.0	30.5	29.5	28.5	32.0
	(Min, Max)	(18, 46)	(18, 49)	(19, 47)	(18, 42)	(21, 49)
**Race, n (%)**						
	White	15 (83.3)	17 (85.0)	13 (65.0)	14 (70.0)	19 (86.4)
	Black or African American	3 (16.7)	3 (15.0)	7 (35.0)	6 (30.0)	2 (9.1)
	Asian	0	0	0	0	1 (4.5)

### 2.2. Safety and Reactogenicity

The HAI-05 vaccine was safe and well tolerated in all groups. No deaths, serious adverse events (SAEs), clinically significant laboratory abnormalities, or AEs of special interest were reported during the trial. No subject withdrew from the study due to an AE (Table 3). Treatment-related AEs that occurred after any vaccination (Table 3) included myalgia in the 15 µg adjuvanted dose group, injection site pain, pruritus, urticaria, back pain, headache and diarrhea in the 90 µg adjuvanted dose group, and pruritus in the 90 µg unadjuvanted dose group.

**Table 3:** Incidence of AEs after any vaccination.

AE category, n (%) ^a^	Adjuvanted HAI-05			Unadjuvanted HAI-05		Placebo
	15 µg	45 µg	90 µg	90 µg		
	N = 18	N = 20	N = 20	N = 20		N = 22
Any AEs	9 (50)	8 (40)	13 (65)	10 (50)		11 (50)
Treatment-related AEs	2 (11.1)	0	4 (20)	1 (5)		0
Serious AEs	0	0	0	0		0
AEs leading to discontinuation from the study	0	0	0	0		0
AEs leading to death	0	0	0	0		0

^a^: Number (%) of subjects reporting symptom at any post-vaccination assessment. Each subject was counted only once.

The incidence of solicited local injection site reactions occurring within 7 days following either vaccination was similar across different adjuvanted HAI-05 dose groups, but was greater than in the unadjuvanted 90 µg dose group and in the placebo group. The most frequently reported reaction was pain at the injection site, with a relatively high incidence rate in the 90 µg adjuvanted dose group after the first dose (80%), which was mild and self-limited. Swelling was infrequent, and no redness was reported in any of the groups following either dose of the vaccine or placebo (Table 4).

The incidence of solicited systemic events occurring within 7 days following either vaccination was similar across different active dose groups and in general was similar to the placebo group. The most frequently reported systemic event was headache, which occurred in all dose groups, but most often in the 90 µg adjuvanted dose group following both the first (35%) and second (30%) vaccination (Table 5).

**Table 4:** Incidence of solicited local injection site reactions after the first andsecond vaccination.

	Adjuvanted HAI-05						Unadjuvanted HAI-05		Placebo	
	15 µg N = 18		45 µg N = 20		90 µg N = 20		90 µg N = 20		N = 22	
**Vaccination n (%) ^a^**	Dose 1 ^b^	Dose 2 ^c^	Dose 1	Dose 2	Dose 1	Dose 2	Dose 1	Dose 2	Dose 1	Dose 2
Pain at Injection Site	11 (61.1)	10 (55.6)	12 (60.0)	11 (55.0)	16 (80.0)	10 (50.0)	1 (5.0)	3 (15.0)	3 (13.6)	1 (4.5)
Redness	0	0	0	0	0	0	0	0	0	0
Swelling	0	1 (5.6)	0	0	2 (10.0)	1 (5.0)	0	0	0	0

^a^: Number (%) of subjects reporting symptom. Each subject was counted only once.

^b^: Between Days 1 and 8.

^c^: Between Days 22 and 29.

**Table 5:** Incidence of solicited systemic events after the first and second vaccination.

	Adjuvanted HAI-05						Unadjuvanted HAI-05		Placebo	
	15 µg N = 18		45 µg N = 20		90 µg N = 20		90 µg N = 20		N = 22	
Vaccinationn (%) ^a^	Dose 1^ b^	Dose 2 ^b^	Dose 1	Dose 2	Dose 1	Dose 2	Dose 1	Dose 2	Dose 1	Dose 2
Fever	0	0	0	0	0	0	0	1 (5.0)	1 (4.5)	0
Tiredness	4 (22.2)	4 (22.2)	4 (20.0)	2 (10.0)	5 (25.0)	1 (5.0)	3 (15.0)	1 (5.0)	2 (9.1)	1 (4.5)
Chills	2 (11.1)	1 (5.6)	1 (5.0)	0	1 (5.0)	0	1 (5.0)	0	2 (9.1)	1 (4.5)
Malaise-Feeling Discomfort	1 (5.6)	2 (11.1)	0	0	3 (15.0)	4 (20.0)	1 (5.0)	0	3 (13.6)	1 (4.5)
Joint Aches	2 (11.1)	2 (11.1)	1 (5.0)	1 (5.0)	1 (5.0)	1 (5.0)	2 (10.0)	0	1 (4.5)	1 (4.5)
Muscle Aches	4 (22.2)	4 (22.2)	3 (15.0)	2 (10.0)	4 (20.0)	3 (15.0)	2 (10.0)	2 (10.0)	2 (9.1)	2 (9.1)
Headache	4 (22.2)	4 (22.2)	4 (20.0)	2 (10.0)	7 (35.0)	6 (30.0)	4 (20.0)	4 (20.0)	4 (18.2)	0

^a^: Number (%) of subjects reporting symptom. Each subject was counted only once.

^b^: Between Days 1 and 8.

^c^: Between Days 22 and 29.

Nearly all AEs were mild to moderate in intensity. The only notable unsolicited AE, localized rash, occurred in one female subject in the 90 µg adjuvanted dose group approximately two days after each vaccination. The subject was receiving chronic topical corticosteroid treatment for psoriasis and a tranquilizer for anxiety before and during the trial. These rash events were not examined or confirmed by the investigator, but were reported by the subject to be mild and resolve without any specific treatment within 48 hours. The subject was lost to follow-up.

Overall, at all dose levels, the HAI-05 vaccine was generally well tolerated, with only mild local injection site reactions which did not appear to be dose related in the presence of Alhydrogel^®^, but were considerably lower without adjuvant even at the highest administered vaccine dose.

### 2.3. Immunogenicity

Mean geometric mean titers (GMTs) of the serum hemagglutination-inhibition (HAI) antibodies for the three adjuvanted dose groups were the same as the mean GMT for the placebo group on Days 1 and 22, and only slightly increased by Day 43. In contrast, the mean GMT for the unadjuvanted 90 µg dose group increased 6.4-fold from Day 1 through 43 (Table 6). Furthermore, seroconversion (10%) after the first vaccination (Day 22) was observed only in the unadjuvanted 90 µg dose group, and after the second vaccination (Day 43) only in the adjuvanted and unadjuvanted 90 µg dose groups (5% and 10%, respectively) (Table 6). The same results were obtained for the proportions of subjects who achieved HAI titers ≥40 (Table 6).

Similar immunogenicity was observed for the adjuvanted dose groups based on mean GMTs of the virus microneutralization (MN) antibodies. In contrast to the adjuvanted dose groups, the mean GMT increased 16-fold from baseline in the unadjuvanted 90 µg dose group after the first vaccination (Days 22) and remained at the same level after the second vaccination (Days 43) (Table 7). At the cut-off titer of ≥20, the unadjuvanted 90 µg dose group was the only active treatment group in which seroconvertion was observed (10%) after the first vaccination (Day 22). However, after the second vaccination (Day 43), some subjects in all of the active dose groups seroconverted in the range from 5.6% to 15% (Table 7). In the post-hoc analysis using a MN antibody cut-off titer of ≥10, 40% of subjects who received the adjuvanted 45 µg vaccine dose seroconverted on Day 43, followed by only 20% of subjects who received the adjuvanted 90 µg dose (Table 7). Also, at the cut-off titer of ≥10 the response rates for the unadjuvanted and adjuvanted 90 µg doses were 15% and 0% on Day 22 and 15% and 20% on Day 43, respectively, further indicating that the presence of Alhydrogel^®^ had no effect on HAI-05 vaccine immunogenicity (Table 7). Surprisingly, one subject (4.5%) in the placebo group (Table 7) who was seronegative at baseline (the titer of 5.0 on Day 1) was also determined as seroconverted after the first vaccination (the titer of 20 on Day 22). The titer on Day 22 was just at the borderline of seropositivity (≥20) and subsequently declined to 7.07 after the second vaccination (Day 43). Furthermore, no seroconversion was observed in other placebo subjects after either the first or the second vaccination, and no subjects in the placebo group seroconverted based on HAI titers (the primary outcome measure). Taken together, these findings suggest that the single placebo subject who appeared to have seroconverted may be as a result of the MN assay variability.

**Table 6:** HAI titers and seroconversion.

Dose of HAI-05	A ^a^	N subjects per group	Pre-vaccination (Day 1)	Post-1st vaccination (Day 22)			Post-2nd vaccination (Day 43)		
			GMT^ b ^(95% CI^ c^)	GMT (95% CI)	Seroconversion n (%) (95% CI)	Titer ≥40 n (%) (95% CI)	GMT (95% CI)	Seroconversion n (%) (95% CI)	Titer ≥40 n (%) (95% CI)
Placebo	–	22	5.0 (5.0, 5.0)	5.0 (5.0, 5.0)	0	0	5.0 (5.0, 5.0)	0	0
15 µg	+	18	5.0 (5.0, 5.0)	5.0 (5.0, 5.0)	0	0	6.41 (4.16, 8.66)	0	0
45 µg	+	20	5.0 (5.0, 5.0)	5.0 (5.0, 5.0)	0	0	6.16 (4.15, 8.18)	0	0
90 µg	+	20	5.0 (5.0, 5.0)	5.0 (5.0, 5.0)	0	0	6.85 (3.83, 9.88)	1 (5.0) (0, 0.1)	1 (5.0) (0, 0.1)
90 µg	–	20	6.75 (3.72, 9.78)	36.50 (0, 73.99)	2 (10.0) (0, 0.2)	2 (10.0) (0, 0.2)	43.23 (0, 89.35)	2 (10.0) (0, 0.2)	2 (10.0) (0, 0.2)

^a^: A, Alhydrogel^®^.

^b^: GMT, geometric mean titer.

^c^: CI, confidence interval.

**Table 7:** MN titers and seroconversion.

Dose of HAI-05	A ^a^	N subjects per group	Pre-vaccination (Day 1)	Post-1st vaccination (Day 22)			Post-2nd vaccination (Day 43)		
			GMT ^b^ (95% CI ^c^)	GMT (95% CI)	Seroconversion n (%) [95% CI]		GMT (95% CI)	Seroconversion n (%) [95% CI]	
					Titer cut-off ≥20	Titer cut-off ≥10		Titer cut-off ≥20	Titer cut-off ≥10
Placebo	–	22	5.78 (4.60, 6.96)	7.37 (4.62, 10.13)	1 (4.5) [0.0, 0.2]	1 (4.5) [0.0, 0.2]	5.53 (4.77, 6.30)	0	0
15 µg	+	18	5.39 (4.88, 5.91)	5.21 (4.83, 5.59)	0	0	8.52 (5.04, 11.99)	1 (5.6) [0.0, 0.2]	3 (16.7) [0.0, 0.3]
45 µg	+	20	5.00 (5.00, 5.00)	5.25 (4.82, 5.68)	0	1 (5.0) [0.0, 0.1]	11.26 (7.41, 15.11)	3 (15.0) [0.0, 0.3]	8 (40.0) [0.2, 0.6]
90 µg	+	20	5.10 (4.92, 5.28)	5.21 (4.96, 5.45)	0	0	11.06 (4.54, 17.58)	2 (10.0) [0.0, 0.2]	4 (20.0) [0.0, 0.4]
90 µg	–	20	8.33 (3.75, 12.91)	132.75 (0, 284.45)	2 (10.0) [0.0, 0.2]	3 (15.0) [0.0, 0.3]	133.77 (0.0, 285.35)	3 (15.0) [0.0, 0.3]	3 (15.0) [0.0, 0.3]

^a^: A, Alhydrogel^®^.

^b^: GMT, geometric mean titer.

^c^: CI, confidence interval.

Taken together, the results of the immunogenicity analyses demonstrate that the experimental HAI-05 vaccine generated low humoral immune responses, with no dose dependence and no enhancing effect of adjuvant.

Currently licensed influenza vaccines, manufactured by several companies globally, are made in embryonated eggs [13]. For the past four decades, these vaccines have been successfully used and were proven to be safe and effective [14,15,16]. Recently, safety and efficacy of several avian H5N1 influenza A vaccines in healthy volunteers have been demonstrated [17,18,19,20,21,22,23]. However, the H1N1 “swine influenza” pandemic of 2009 demonstrated that egg-based technologies do not have the capacity to satisfy the global need for an emerging pandemic influenza vaccine in a timely manner. Therefore, a number of countries, including the U.S., are making significant investments in developing alternative technologies that could satisfy this unmet need.

In the last several decades, an alternative, recombinant protein-based approach has been introduced to vaccine development and manufacturing. In contrast to conventional vaccines, the recombinant, or subunit, vaccines represent individual immunogenic proteins derived from target pathogens. The subunit vaccine approach is, therefore, considered relatively safe compared to vaccination with whole organism and can be easily scaled up to meet healthcare needs. Some of these alternative manufacturing approaches are based on mammalian and insect cell expression systems for the production of recombinant proteins, including influenza vaccines [24,25,26,27,28,29,30,31].

Over the past ten years, plants have emerged as a highly promising approach to economically manufacture recombinant proteins, including vaccine antigens [32,33,34,35]. There have been numerous pre-clinical studies demonstrating the immunogenicity and protective efficacy of plant-produced vaccine candidates against a variety of pathogens [1,2,9,10,11,36]. More recently, several groups have tested plant-produced vaccines and therapeutic products in humans and demonstrated safety and biological relevance of these products following parenteral administration in Phase 1-3 clinical trials [37,38,39,40,41].

We have engineered and produced HAI-05, a recombinant HA antigen from A/Indonesia/05/2005 (H5N1) strain of influenza A virus in a “launch vector”-based transient expression system. This was achieved in less than one month and was subsequently scaled up for cGMP manufacturing at FhCMB’s pilot plant facility [2]. Following successful pre-clinical evaluation, including a Good Laboratory Practice-compliant toxicology study in a rabbit model [2], the experimental vaccine entered into a Phase 1 human study. 

The vaccine was tested at 15 and 45 µg doses with Alhydrogel^® ^adjuvant and at 90 µg dose with and without Alhydrogel^®^ adjuvant and administered in a two-dose regimen three weeks apart. At these doses, the vaccine was generally shown to have a favorable safety profile in healthy volunteers, with no reported SAEs and no evidence of any dose-limiting or dose-related toxicity. As expected, the most frequent event was local injection site reaction after either dose, which was generally mild and self-limited.

Despite the positive immunogenicity results in the pre-clinical studies [2], immune responses elicited by the HAI-05 vaccine in this study were low and somewhat variable with respect to both HAI and MN antibody titers, with the highest responses observed in the 90 µg unadjuvanted group, indicating no enhancement in the presence of Alhydrogel^®^ adjuvant. By contrast, in pre-clinical studies, Alhydrogel^®^ enhanced antibody responses elicited by HAI-05 and provided a dose-sparring effect in animal models [2]. In addition, in a randomized, Phase 1 clinical trial, alum-adjuvanted whole-virus avian A/Hong Kong/1073/99 (H9N2) vaccine was shown to be more immunogenic than unadjuvanted vaccine [42]. The trial results reported here are consistent with findings from other randomized, controlled influenza vaccine clinical studies in different populations of human volunteers. Indeed, several groups evaluating split-virion 2009 pandemic influenza A H1N1 vaccines [43,44,45] as well as split-virion or whole-virus influenza A H5N1 vaccines have reported that alum either failed to enhance or even decreased antibody production [18,20,46,47,48,49,50]. The reason for Alhydrogel^®^’s inefficiency is not known, since potency of this adjuvant in stimulating both T helper 1 and 2 type responses and antibody production has been well documented [51]. As suggested in a previous study by Liang *et al.* [43], the absence of benefit may be due to delayed antigen release from alum-adjuvanted formulations.

The low immunogenicity observed in this study is likely attributable to the use of a suboptimal dose of the H5 HA antigen in the HAI-05 vaccine. A post-hoc analysis of MN antibody responses using a cut-off titer of ≥10 revealed responses that were high among responders, which is suggestive of a threshold-like effect that may be further enhanced. Furthermore, the results of a completed Phase 1 trial of plant-produced HAC1 influenza vaccine (recombinant HA derived from A/California/04/2009 [H1N1] strain of influenza A virus) have demonstrated that HAC1 is immunogenic when compared with a licensed, egg-derived H1N1 vaccine [52]. Therefore, and as further supported by studies of conventional egg-derived H5N1 vaccines and recombinant H5N1 vaccines produced in other expression systems, the underwhelming results observed with the HAI-05 vaccine in this study appear to be related to the H5 antigen itself.

## 3. Experimental Section

### 3.1. Study Design

This study was a first-in-human, Phase 1 randomized, double-blind, placebo-controlled, dose-escalation clinical study conducted at two clinical centers in the U.S. The study was conducted in accordance with the Declaration of Helsinki and the Code of Federal Regulations of the United States Food and Drug Administration (FDA) and in compliance with the International Conference on Harmonization Guidelines for Good Clinical Practice. The study protocol, informed consent form and subject recruitment procedures were reviewed and approved by an Institutional Review Board and an Independent Ethics Committee. All participants provided written informed consent prior to screening and enrollment into the study.

The primary objective of the study was to evaluate the safety, reactogenicity and tolerability of three escalating dose levels of the HAI-05 vaccine—15, 45 and 90 µg adjuvanted with Alhydrogel^®^ and 90 µg without Alhydrogel^®^—and placebo (0.9% normal saline) delivered intramuscularly to healthy adults 18–49 years of age. The secondary objective was to evaluate and compare immunogenicity of these four HAI-05 vaccine formulations after two doses based on HAI and MN antibody titers.

This study was registered under clinical trial reference identifier NCT01250795 [53].

### 3.2. Vaccine

The HAI-05 vaccine is a recombinant subunit HA antigen from the A/Indonesia/05/2005 (H5N1) strain of influenza A virus. The HA sequence encompassing amino acids 17–532 was optimized for expression in plants and synthesized by GENEART AG (Regensburg, Germany). To obtain the truncated HA molecule in the plant expression system, the transmembrane domain (a.a. 533–569) and signal peptide (a.a. 1–16) were removed from the entire HA sequence and the pathogenesis-related protein 1a (PR-1a) signal peptide was added to the N-terminus [1]. A poly-histidine (6 × His) affinity purification tag followed by the endoplasmic reticulum retention signal (KDEL) were added to the C-terminus [1]. The HA antigen has been cloned, expressed in *N. benthamiana*, and purified as described previously [2]. The purified HAI-05 protein has a monomeric solution state with a purity of >90% as determined by SDS-PAGE and reverse-phase chromatography [2]. Glycosylation analysis, as reported previously, indicated that the core glycan structure was followed by high mannose attachments as expected with use of the KDEL retention sequence. All six potential N-linked glycosylation sites (Asn10, Asn23, Asn154, Asn165, Asn286 and Asn484) showed the high mannose glycosylation [2].

The concentration of the HAI-05 formulated drug substance was 360 μg/mL in a formulation of 0.9% normal saline (United States Pharmacopeia, Hospira, Lake Forrest, IL) with trace amounts of phosphate buffered saline (PBS: 50 mM NaCl, 0.7 mM KCl, 3.6 mM Na_2_HPO_4_, and 0.7 mM KH_2_PO_4_) for intramuscular administration.

### 3.3. Study Population and Treatment

Healthy male and non-pregnant female adults 18–49 years of age were excluded from enrollment if they received prior vaccination with any influenza vaccine containing H5 antigen; had any medical condition that may be associated with impaired immune responsiveness, including diabetes mellitus, cancer or treatment for cancer within the previous three years, excluding basal cell carcinoma or squamous cell carcinoma; had a history of anaphylactic type reaction to injected vaccines, positive serology for human immunodeficiency virus (HIV) types 1 and 2 (HIV-1 and HIV-2), hepatitis B surface antigen or hepatitis C virus antibodies or any acute or chronic pulmonary, cardiovascular, hepatic, neurologic or renal disease that might confound evaluation of the vaccine; or had recently taken or planned to take any other experimental vaccine within 30 days prior to vaccination. 

A total of 100 subjects were enrolled and randomized into 4 cohorts, each with 20 subjects to receive the HAI-05 vaccine formulation and 5 subjects to receive placebo (4:1 vaccine to placebo ratio) (Table 8). Subjects in the study vaccine groups received two doses of HAI-05 at 15, 45 or 90 μg administered with 0.3% aluminum hydroxide (Alhydrogel^®^) adjuvant (Brenntag Biosector, Denmark) or at 90 μg administered without adjuvant. Subjects in the placebo control group received two doses of saline (0.9% sodium chloride, United States Pharmacopeia). All doses were administered in a volume of 0.5 mL.

Vaccine was administered in the deltoid muscle of the same non-dominant arm on Days 1 and 22. Progression to a next higher dose was determined by a Cohort Review Committee based on the safety of a lower dose and in accordance with specified halting rules. After the 15 and 45 μg dose levels were determined to be well tolerated, the two 90 μg dose levels (with and without Alhydrogel^®^) were administered simultaneously to subjects in the respective cohorts.

**Table 8:** Study cohorts and treatments.

Cohort	HAI-05 treatment			Placebo treatment	
	Number of subjects	Dose of HAI-05 (µg/0.5 mL)	Dose of Alhydrogel^® ^(mg aluminum/0.5 mL)	Number of subjects	Placebo
1	20	15	0.75	5	Saline
2	20	45	0.75	5	Saline
3	20	90	0.75	5	Saline
4	20	90	–	5	Saline

### 3.4. Safety Assessments

Safety assessments (primary endpoints) consisted of reactogenicity events, AEs, clinical laboratory tests (serum chemistry, hematology and urinalysis), vital signs, and abnormal findings upon physical examination. 

At 30 minutes after each dosing (Days 1 and 22), any signs or symptoms of local and systemic reactions to the study medications (immediate complains) were recorded in an electronic Case Report Form. In addition, vaccinees were instructed to record any occurring reactogenic events for 7 days after each dosing (between Days 1 and 8 and Days 22 and 29, respectively) in a Subject Diary. The solicited immediate complaints and reactogenic events included: pain, redness or swelling at the injection site; fever; muscle or joint aches; headache; tiredness; chills; and malaise (feeling of discomfort or uneasiness). These symptoms were not recorded as AEs unless they were SAEs or persisted beyond Days 8 or 29. Any unsolicited symptoms (AEs) were recorded at 30 minutes after vaccination by the clinical site staff, and thereafter through Day 43 by subjects in a Supplemental Diary. 

A SAE was defined as any untoward medical occurrence that resulted in death, was life threatening, resulted in persistent or significant disability/incapacity, required hospitalization or prolongation of existing hospitalization, or was associated with a congenital abnormality/birth defect in the offspring of a study subject. The occurrence of any SAE was monitored throughout the course of the study. 

AEs were assessed for intensity using standardized criteria adapted from the September 2007 FDA Guidance entitled “Toxicity Grading Scale for Healthy Adult and Adolescent Volunteers Enrolled in Preventive Vaccine Clinical Trials” [54]. The intensity was determined as mild, moderate or severe, based on the definitions provided in the Guidance. Vaccine-related AEs were those that the investigator judged as having a reasonable possibility that the vaccine contributed to the AE. AEs were coded to the Medical Dictionary for Regulatory Activities^®^ (MedDRA) system organ class and preferred terms.

### 3.5. Immunogenicity Assessments

Sera for immunogenicity assessments were collected on Days 1 (pre-vaccination; baseline), 22 (pre-vaccination; results for the first vaccination) and 43 (results for the second vaccination), and analysed for HAI and MN antibody titers against A/Indonesia/05/2005 (H5N1) using validated assays at Southern Research Institute (Birmingham, AL).

Immunogenicity assessments included determination of 1) GMTs of HAI and MN antibody titers; 2) the proportion of subjects in each group who seroconverted on Days 22 and 43 based on HAI and MN antibody titers; and 3) the proportion of subjects in each group who achieved HAI titers ≥40 on Days 22 and 43. For HAI titers, seroconversion was defined according to the FDA guidance as the percentage of subjects with either a pre-vaccination titer <10 and a post-vaccination titer ≥40 or a pre-vaccination titer ≥10 and a minimum 4-fold rise in a post-vaccination antibody titer. For MN titers, seroconversion was defined as the percentage of subjects with either a pre-vaccination titer at the limit of detection at the starting dilution of 1:20, designated to have a titer of 5, and a post-vaccination titer ≥20, or a pre-vaccination titer ≥20 and a minimum 4-fold rise in a post-vaccination antibody titer. In a post-hoc analysis of MN seroconversion, a post-vaccination MN titer of ≥10 was used as a cut-off value.

### 3.6. Statistical Analyses

Statistical analyses were performed by PharmaNet Development Group, Inc. (Princeton, NJ). Safety analyses were based upon the intent-to-treat population defined as all volunteers who received at least one dose of vaccine or placebo and one post-vaccination visit. Immunogenicity analyses were based upon the per-protocol population defined as all subjects who received two doses of vaccine and who had provided serum specimens for antibody determinations. 

All statistical analyses were performed using SAS^®^ Version 8.2 or higher. Categorical data were summarized using counts and percentages. Unless otherwise specified, descriptive statistics included: n, mean, standard deviation (SD), median, coefficient of variation (CV%), and minimum and maximum for continuous variables. In general, minimum and maximum were quoted to the number of decimal places as recorded in the electronic case report form; means, medians, SD, and CV% were quoted to one (1) further decimal place. Percentages were rounded to one decimal place. All statistical tests were two-sided and were performed using a 5% significance level, unless otherwise stated, leading to 95% (two-sided) confidence intervals (CIs).

Demographic data of vaccinated subjects were summarized by treatment and analyzed by descriptive statistics. All AEs were summarized by numbers and percentages of subjects with corresponding AEs. Proportions of subjects in each treatment group with AEs within each body organ system were compared in the same manner. Immediate complaints and reactogenicity events were summarized by number of events and the percentage, and each dose group value was compared to the placebo group value using Fisher’s exact test. Clinical laboratory results were summarized using descriptive statistics.

Proportions of subjects who developed a significant rise in antibody titers after the first dose (secondary endpoint) and second dose (primary endpoint) in each dose group were determined using standard statistical analyses. The HAI and MN GMTs and their 95% CIs were summarized for the two vaccination days (Days 1 and 22) and post-second dose (Day 43) with stratification by cohort. Point estimates and the two-sided 95% CIs for these evaluations were summarized for seroconversion and subjects achieving titers ≥40 (for HAI) and for seroconversion (for MN) for the two vaccination days (Days 1 and 22) with stratification by cohort, along with the post-second dose (Day 43) results.

## 4. Conclusions

The results of this first-in-human, Phase 1 randomized, double-blind, placebo-controlled, dose-escalation clinical study demonstrated that the experimental HAI-05 vaccine was generally safe at all dose levels. Mild local injection site reactions occurred across all dose levels, but at a considerably lower incidence rate in the absence of Alhydrogel^® ^ adjuvant. The vaccine generated low humoral immune responses in healthy adult subjects, with no dose dependence and no enhancing effect of adjuvant, suggesting the use of a suboptimal dose of the H5 HA antigen.
